# A Closed-Loop Falls Monitoring and Prevention App for Multiple Sclerosis Clinical Practice: Human-Centered Design of the Multiple Sclerosis Falls InsightTrack

**DOI:** 10.2196/49331

**Published:** 2024-01-11

**Authors:** Valerie J Block, Kanishka Koshal, Jaeleene Wijangco, Nicolette Miller, Narender Sara, Kyra Henderson, Jennifer Reihm, Arpita Gopal, Sonam D Mohan, Jeffrey M Gelfand, Chu-Yueh Guo, Lauren Oommen, Alyssa Nylander, James A Rowson, Ethan Brown, Stephen Sanders, Katherine Rankin, Courtney R Lyles, Ida Sim, Riley Bove

**Affiliations:** 1 Department of Neurology University of California San Francisco Weill Institute University of California San Francisco San Francisco, CA United States; 2 Department of Physical Therapy and Rehabilitation Science University of California San Francisco San Francisco, CA United States; 3 University of California San Francisco Division of General Internal Medicine Zuckerberg San Francisco General Hospital San Francisco, CA United States; 4 Center for Vulnerable Populations University of California San Francisco San Francisco, CA United States; 5 Department of Medicine University of California San Francisco San Francisco, CA United States

**Keywords:** digital health, mobile tools, falls, prevention, behavioral medicine, implementation science, closed-loop monitoring, multiple sclerosis, mobile phone

## Abstract

**Background:**

Falls are common in people with multiple sclerosis (MS), causing injuries, fear of falling, and loss of independence. Although targeted interventions (physical therapy) can help, patients underreport and clinicians undertreat this issue. Patient-generated data, combined with clinical data, can support the prediction of falls and lead to timely intervention (including referral to specialized physical therapy). To be actionable, such data must be efficiently delivered to clinicians, with care customized to the patient’s specific context.

**Objective:**

This study aims to describe the iterative process of the design and development of Multiple Sclerosis Falls InsightTrack (MS-FIT), identifying the clinical and technological features of this closed-loop app designed to support streamlined falls reporting, timely falls evaluation, and comprehensive and sustained falls prevention efforts.

**Methods:**

Stakeholders were engaged in a *double diamond* process of human-centered design to ensure that technological features aligned with users’ needs. Patient and clinician interviews were designed to elicit insight around ability blockers and boosters using the capability, opportunity, motivation, and behavior (COM-B) framework to facilitate subsequent mapping to the Behavior Change Wheel. To support generalizability, patients and experts from other clinical conditions associated with falls (geriatrics, orthopedics, and Parkinson disease) were also engaged. Designs were iterated based on each round of feedback, and final mock-ups were tested during routine clinical visits.

**Results:**

A sample of 30 patients and 14 clinicians provided at least 1 round of feedback. To support falls reporting, patients favored a simple biweekly survey built using REDCap (Research Electronic Data Capture; Vanderbilt University) to support *bring-your-own-device* accessibility—with optional additional context (the severity and location of falls). To support the evaluation and prevention of falls, clinicians favored a clinical dashboard featuring several key visualization widgets: a longitudinal falls display coded by the time of data capture, severity, and context; a comprehensive, multidisciplinary, and evidence-based checklist of actions intended to evaluate and prevent falls; and MS resources local to a patient’s community. In-basket messaging alerts clinicians of severe falls. The tool scored highly for usability, likability, usefulness, and perceived effectiveness (based on the Health IT Usability Evaluation Model scoring).

**Conclusions:**

To our knowledge, this is the first falls app designed using human-centered design to prioritize behavior change and, while being accessible at home for patients, to deliver actionable data to clinicians at the point of care. MS-FIT streamlines data delivery to clinicians via an electronic health record–embedded window, aligning with the *5 rights* approach. Leveraging MS-FIT for data processing and algorithms minimizes clinician load while boosting care quality. Our innovation seamlessly integrates real-world patient-generated data as well as clinical and community-level factors, empowering self-care and addressing the impact of falls in people with MS. Preliminary findings indicate wider relevance, extending to other neurological conditions associated with falls and their consequences.

## Introduction

### Background

Falls are common in patients with multiple sclerosis (MS), occurring in 50% to 70% of published cohorts, a rate similar to that of older adults [1]. Falls often lead to injury, result in significant health care costs [2-5], and increase *the* *fear of falling* [6,7]; furthermore, they lead to a decline in physical activity and participation in daily life as well as cause loss of independence [8,9]. Targeted interventions such as physical therapy (PT) can reduce falls and the fear of falling [10-12], but patients often underreport and clinicians undertreat this issue. Indeed, fewer than half of the people with MS who report falls receive falls prevention information from their clinician [13], and there is a lack of self-management apps to engage and empower people with MS about falls prevention [14-16].

To address this gap, multimodal closed-loop tools hold promise. Closed-loop tools can use real-time feedback and patient-generated data (PGD; such as those already validated in MS [17-22]) to continuously monitor and adjust interventions to improve outcomes. Such an approach has been used in biological functions and symptoms, such as insulin delivery or depression [23-25]. Unfortunately, in MS, apps on the commercial market exist outside of the health system, that is, away from the point of care. To close these gaps in care, a tool should close the loop of information flow from the patient to the appropriate clinician (depending on the diagnosis and symptoms being treated, ie, neurologist) at the point of care and back to the patient to support patient-centered care. Furthermore, the tool must address the behavioral barriers to change to promote the behaviors (eg, reporting, screening, treatment recommendations, and follow-up with timely refills or referral scheduling) likely to lead to falls prevention. From previous work, real-time PGD such as prospective near-falls reports, patient-reported outcomes, and changes in step count captured by wearable sensors all provide useful input for the closed-loop models [26,27]. The integration of these in a multimodal tool would enhance falls prediction accuracy and could act as an early warning system for timely PT referrals, reducing falls risk and related injuries [28,29]. However, challenges lie in delivering PGD to the point of care, granting access for prompt intervention, and active self-management. To be actionable, these PGD, generated from remote devices or patient-reported outcomes, must be delivered according to the *5 rights* [30]: the right information, to the right person, in the right format, through the right channel, at the right time in the workflow. This is a hurdle that health systems have for the most part not yet overcome, and PGD are not typically integrated into care systems.

To address these challenges, we developed Multiple Sclerosis Falls InsightTrack (MS-FIT), a closed-loop falls monitoring and prevention app. MS-FIT enables seamless information exchange between patients and clinicians, driven by stakeholder input and human-centered design (HCD) principles [31,32]. It empowers individuals with MS to *track* falls, enhances clinician decision-making by providing real-world *insights*, and fills a crucial gap in self-management for falls monitoring and prevention.

### Objectives

This paper describes the iterative process of the design and development of MS-FIT. MS-FIT is designed to integrate various data types to personalize falls risk assessments and interventions for individuals with MS. To achieve this, a planned process of engagement of patients and clinicians (ie, neurologists) was performed to ensure that MS-FIT aligns with user needs, whereas usability evaluations validated its potential impact on falls prevention. Subsequently, we will test the feasibility of implementation and effectiveness of MS-FIT in a larger clinical trial.

## Methods

### Study Setting

The primary clinical setting is the University of California San Francisco (UCSF) Multiple Sclerosis and Neuroinflammation Center, which provides specialized care to >6000 adults with MS annually. Clinician stakeholders were approached via email or in person and invited to participate in the study. Patients who had given permission to be contacted for research participation or who had sustained falls in the past year were invited via secure email to participate as stakeholders.

### Ethical Considerations

The University of California San Francisco Institutional Review Board approved all study activities (22-36680). Informed consent forms and Health Insurance Portability and Accountability Act documents were signed by each study participant (patients, clinicians, and other interviewees). Patients received US $50 (1-time compensation) for their participation in the study.

### Study Design

The overarching approach was grounded in the principles and phases of HCD [31]. This process focuses on the usability and needs of those whom the tool is meant to serve, in this case, patients and clinicians. The development protocols included (1) thorough engagement from a comprehensive range of stakeholders, (2) models based on HCD approaches to ensure alignment with the needs of the intended users (patients and clinicians), (3) an evaluation of the tool’s usability using an established framework: the Health IT Usability Evaluation Model (Health-ITUEM) [33], and (4) plans to support the generalizability and scalability of the tool to other clinical settings associated with falls.

**Figure 1:**
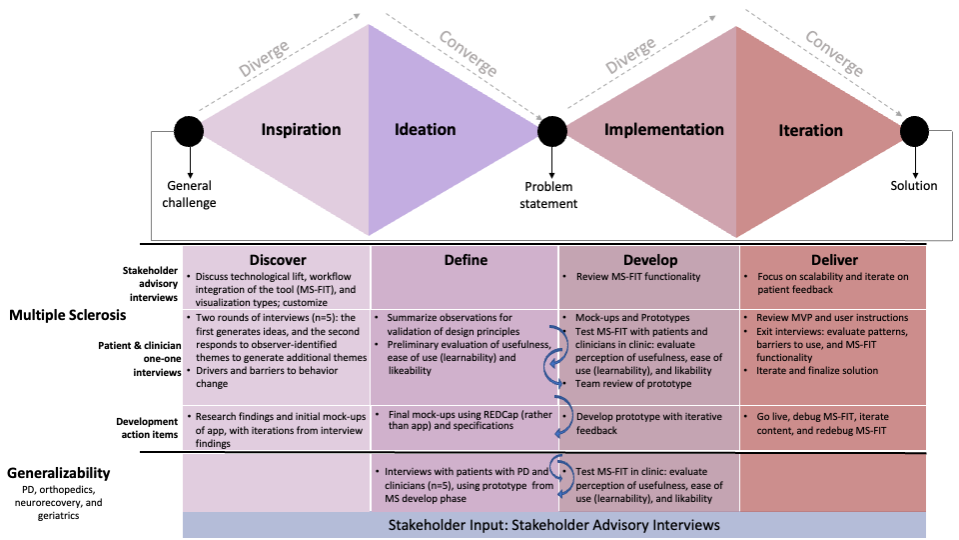
Modified double diamond approach: phases of development and stakeholder engagement. The double diamond depicts the human-centered design principles and framework, with iterations through the discover, define, develop, and delivery phases. The timeline and workflow of the human-centered design phases depict corresponding interviews and products. The curved arrows between “Define” and “Develop” indicate an iterative process between these 2 phases. MS: multiple sclerosis; MS-FIT: Multiple Sclerosis Falls InsightTrack; MVP: minimum viable product; PD: Parkinson disease; REDCap: Research Electronic Data Capture.

**Figure 2:**
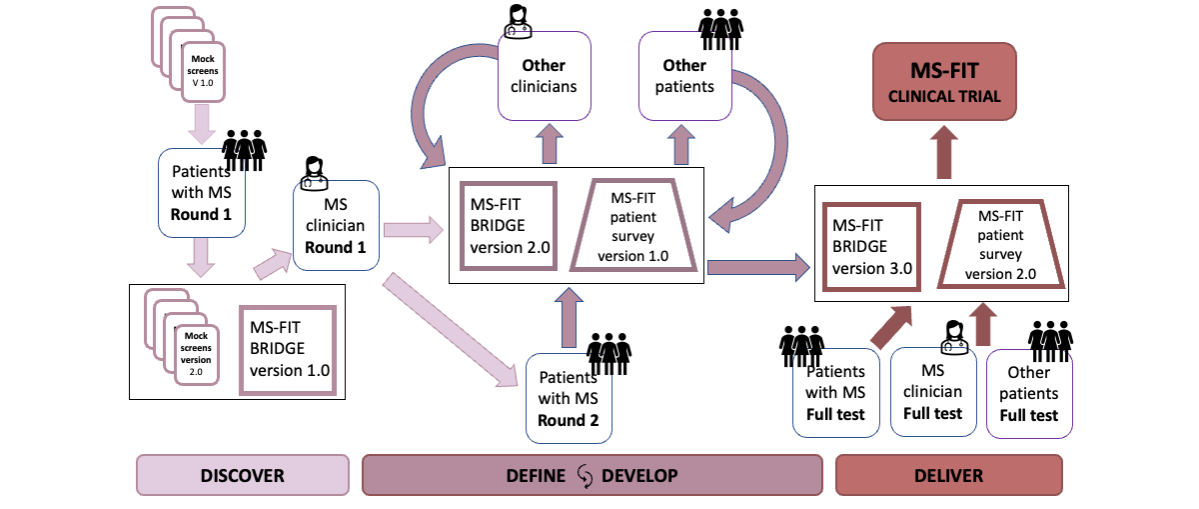
The trajectory of Multiple Sclerosis Falls InsightTrack (MS-FIT) though the phases of development and stakeholder engagement. The final tool components include a patient survey (MS-FIT patient survey) and a clinical dashboard (MS-FIT BRIDGE). The trajectory integrates feedback from user (patient and clinician) interviews through the phases of discover, define, develop (iterative), and deliver. The version numbers indicate a revised version of the patient- or clinician-facing prototype. “Other patients” refers to patients with Parkinson disease as well as orthopedics, neurorecovery, and geriatrics populations. “Full test” refers to the prototype testing in the contextual environment. MS: multiple sclerosis.

HCD involves a series of steps, articulated initially in the context of design [34] and expanded to health care [35]: inspire (empathize with all stakeholders), ideate (define the problem and conceptualize in an open-minded manner), implement (prototype solutions and test), and iterate. Figure 1 illustrates these phases in a *modified double diamond* approach as they were undertaken in the current project, depicting the iterative broadening and narrowing of content and layout throughout the phases [36]. Figure 2 shows the trajectory of MS-FIT and the assimilation of insights obtained from user interviews (involving patients and clinicians) throughout the phases of discover, define, develop (iterative), and deliver.

**Figure 3:**
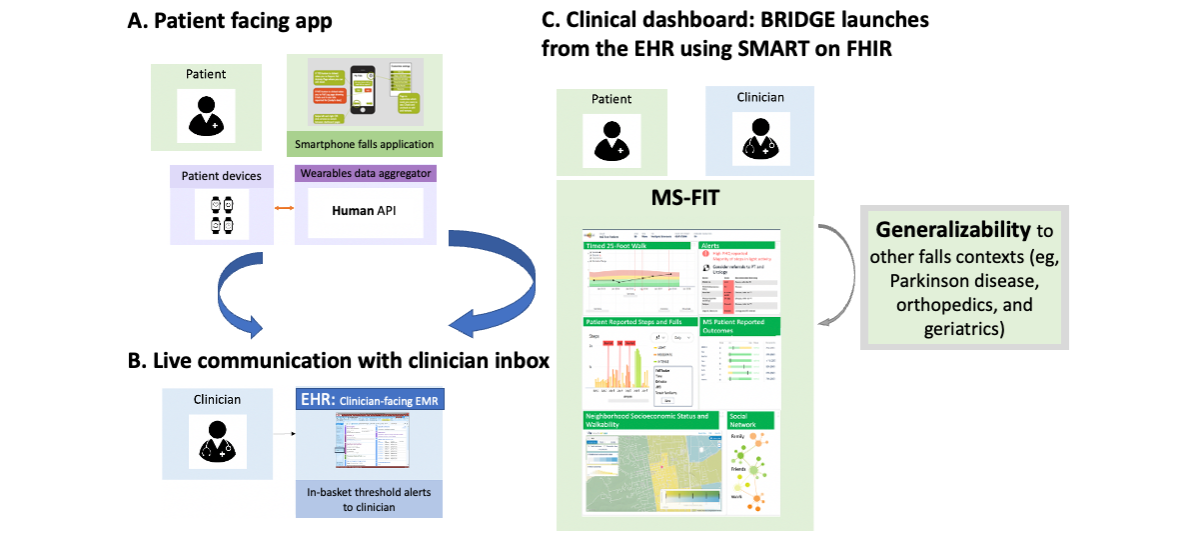
Initial proposal for Multiple Sclerosis Falls InsightTrack (MS-FIT), which involved designing a closed-loop integrated MS-FIT personal health library. MS-FIT is designed to enable patients to track their falls in the context of their lived experience, report them to their care team, and gain insight into multimodal contributors to falls, falls’ impact on daily life participation, and interventions likely to prevent falls. Clinicians, by using BRIDGE, can gain insight into which patients are falling between clinical encounters and how best to personalize risk reduction interventions for the individual patient. This prototype was generated from a number of insights from observational and interventional studies that used patient-generated data to monitor walking and falls in people with multiple sclerosis (MS) and from clinician-facing [42] and patient-facing [43] apps designed using human-centered design to facilitate shared decision-making and evidence-based practice in MS. (A) Patient facing app; (B) Live communication with clinician inbox; (C) Clinical dashboard: BRIDGE launches from the EHR using SMART or FHIR. API: application programming interface; EHR: electronic health record; EMR: electronic medical record; FHIR: Fast Healthcare Interoperability Resources; MS-FIT: Multiple Sclerosis Falls InsightTrack; SMART: Substitutable Medical Apps and Reusable Technologies.

The initial prototype (prediscover) was developed based on feedback from extensive HCD of the BRIDGE point-of-care clinical dashboard (refer to the Technological Building Blocks subsection) summarized elsewhere [37,38], where both patients and clinicians expressed a desire for the integrations of features and episodes of falls to be incorporated into the design. The study team initially identified key elements for MS-FIT through a combination of clinical expertise and literature review [39,40] (Figure 3). These elements were then amalgamated into mock app screens using PowerPoint (Microsoft Corp) for the first round of patient interviews. Figure 3 illustrates the inaugural prototype, which was informed by valuable insights from observational [41] and interventional [39] studies that used PGD to monitor walking and falls in individuals with MS. In addition, the prototype draws inspiration from clinician-facing [42] and patient-facing [43] apps designed using HCD principles to promote shared decision-making and evidence-based practice in MS.

### Framework for Tool Evaluation

The Health-ITUEM framework appraises both subjective and objective outcomes that inform a tool’s usability [44]. In the design phases described herein, the subjective outcomes (satisfaction measured by the perceived ease of use and perceived usefulness) were primarily evaluated. Furthermore, the 4 key variables proposed by Mathews et al [45] to determine both (1) whether the tool (MS-FIT) reflects HCD principles and (2) whether it is likely to engage patients were applied. These four domains encompass (1) usefulness, (2) ease of use or learnability, (3) likability, and (4) effectiveness. These frameworks were used to categorize critical data and visualization elements, as well as the technological and clinical workflow aspects of MS-FIT [46].

### Technological Building Blocks

The architecture of the tool was built leveraging existing tools, primarily BRIDGE and REDCap (Research Electronic Data Capture; Vanderbilt University).

#### BRIDGE

The BRIDGE precision medicine platform at UCSF is an application programming interface (API) that assembles clinical and research data from a variety of sources into a dashboard customized for a given clinical context, displaying a series of digestible, actionable visualizations [38]. BRIDGE is integrated with the Epic electronic health record (EHR; Epic Systems Corporation), launches from Epic using Substitutable Medical Apps and Reusable Technologies (SMART) on Fast Healthcare Interoperability Resources (FHIR; a standard approach for building reusable and extendable EHR-integrated apps), and is integrated with Epic FHIR APIs and other data integrations. The back-end of BRIDGE is built using Python, the flask framework, and PostgreSQL to store configuration data. Although individual-level data will populate the tool, cohort-level data can become the reference cohort against which an individual’s data can be contextualized. BRIDGE pulls data not only from the EHR but also from a range of custom research databases as well as other APIs, such as REDCap [38]. BRIDGE was developed based on extensive HCD processes both within the field of MS [42,43] and beyond [37]. The data visualizations can be developed using HTML, cascading style sheets (CSS), JavaScript, Data-Driven Documents–JavaScript, and other front-end libraries. Each front-end visualization is modular, allowing for asynchronous loading, and is a parameterized JavaScript component, allowing us to extend the code to additional platforms and data sources. Data formatting standards are also applied to make all visualizations and data inputs modular. All API calls are made in real time; BRIDGE does not store patient data, but there is an option to write back to the EHR by pasting the visuals into a clinical note. Furthermore, the development team follows universal design principles, influenced by the Agency for Healthcare Research and Quality Toolkit for Designing Consumer Health IT [47].

#### REDCap Tool

REDCap [38] includes editable or annotatable functions to enable patients to keep track of, and annotate, their PGD. Design choices reflect digital health literacy principles and feedback provided from diverse patients. Together, these enhancements make the data understandable and actionable.

### Investigator Team

The core team included an MS neurologist with HCD expertise (RB), software engineers (NM and NS), a health literacy and patient engagement expert (JR), and an MS physical therapist with remote ambulatory and falls monitoring expertise (VJB). Additional key scientific input was provided by a digital health cloud infrastructure expert (IS), an implementation science expert (CL), a health disparities and population health expert (CL), and an expert in large-scale mobile health (IS). Patient stakeholders included National Multiple Sclerosis Society advocates (LG) and patients (3 core stakeholders). Research team members included a program manager (KK) and clinical coordinators (JW and KH). Before starting the project, this team met to determine the phases of research and design an initial mock-up of the tool that could be used during the discover phase. Volunteer consultants included a software engineer (JR) and user interface or user experience experts.

### Phases of Design

#### Phase 1: Discover

##### Stakeholder Advisory Team

An initial stakeholder meeting took place, during which the goals and phases of the project were outlined. Next, the core team met biweekly as a group or as subgroups to discuss an agenda that included the development of patient and clinician interview guides, interview coding schemes and thematic analysis, the practical aspects of the technological lift, the workflow integration of MS-FIT, and the visualization types and customizations. The iterations of mock-ups were revised based on patient and clinician interview feedback.

##### Interviews

One-on-one interviews were conducted by the health literacy and patient engagement expert (hereinafter referred to as the interviewer) with patients (round 1) and clinicians. Because of ongoing COVID-19 restrictions on in-person engagements, interviews were conducted via the UCSF Zoom video platform (Zoom Video Communications, Inc) using interview guides developed for each audience to elucidate how a tool might be designed to promote behavior change around falls ascertainment, reporting, and prevention. All questions were administered verbally, and interviews lasted between 45 and 60 minutes. With participant consent, interviews were simultaneously recorded and transcribed using Zoom’s video transcription feature.

Interview guides included qualitative and quantitative components. Open-ended questions probed around the domains of the capability, opportunity, motivation, and behavior (COM-B) framework to facilitate subsequent mapping to the Behavioral Change Wheel (BCW) proposed by Michie et al [48]. Quantitative questions with Likert-style responses (ranging from 1=lowest to 5=highest) were administered verbally throughout each interview to assess specific aspects of patient and clinician experience related to capability, opportunity, and motivation, as well as the perceived usefulness of mock screen views and workflows. Participants were asked to comment on their Likert-style responses.

Patient interviews were semistructured around 2 key thematic topics: patient experience with (1) falls and activity, including ability to be active, knowledge, communication with care team, experience, feelings, and expectations; and (2) use of technology, including smartphone, tracking devices, apps, and communication with care team. To complement qualitative insights, patients were asked to use a Likert scale to rate the perceived usefulness of each of 3 app screen views featuring different design elements.

Semistructured interviews with clinicians started with a review of the activity blockers and boosters identified during the discover phase interviews with patients. With this insight, clinicians were asked a series of open-ended qualitative questions to elicit their perspectives on whether a falls reporting tool might promote sustainable falls prevention, as well as gather feedback on the initial closed-loop design (Figure 3) intended to support falls treatment and clinical decision support. To assess each design feature, clinicians were asked to rate perceived usefulness on a Likert scale.

##### Analysis

After all interviews were concluded for each audience, the interviewer reviewed each transcript and used inductive coding to develop a coding scheme on the basis of responses to the open-ended questions [46]. Frequently occurring topics were assigned a unique thematic category, and less frequent topics were coded *other*. Categories were defined by the interviewer, and quotations from the transcript were used to illustrate the type of text coded into the category. Although the interviewer was the sole coder, the stakeholder advisory team provided ongoing consultation on the coding scheme and how to code less frequently occurring responses.

The interviewer transferred Likert-style response data to a spreadsheet to calculate means and SDs for each question. To analyze questions designed to map to the COM-B framework, the interviewer created a data grid where the rows were COM-B categories with subthemes of ability blocker and booster types, and the columns were evidence (quotes) of specific blockers or boosters [49]. Evidence of blockers or boosters that spanned >1 category were placed in all relevant categories to ensure that they would be represented when considering BCW-guided interventions.

After developing the initial COM-B data grid, the interviewer, in consultation with the stakeholder advisory group, expanded the grid to include (1) BCW intervention functions to help users overcome barriers to performing target behaviors and (2) potential intervention solution features designed to be effective for each corresponding blocker category. Intervention solution features were subsequently added to the design road map for immediate or future implementation.

#### Phases 2 and 3: Define and Develop (Iterative)

##### Stakeholder Advisory Team

In these phases, the team reviewed qualitative and quantitative findings from additional patient (2 rounds) and clinician (1 round) interviews and used this feedback to further refine MS-FIT tool functionality, including design and technological features. Changes were prioritized according to the strength of feedback (occurrence of themes and usability scores) and technical feasibility.

##### Interviews

The define and develop phases encompassed a second round of patient interviews, followed by 2 rounds of interviews with clinicians and patients designed to assess MS-FIT generalizability to other high-risk clinical contexts. The same process was followed as that described in phase 1 (discover). One-on-one interviews were conducted by the interviewer via the UCSF Zoom video platform using interview guides. All questions were administered verbally, and interviews lasted between 45 and 60 minutes. With participant consent, interviews were simultaneously recorded and transcribed using Zoom’s video transcription feature.

##### Patient Interviews (Round 2)

Interview guides included qualitative and quantitative components. In an effort to validate the patient experience findings from round 1 interviews, patients interviewed during round 2 were similarly asked to share qualitative feedback around personal experiences with falls, falls and near-falls reporting, perceived benefits and concerns around using a falls tracking app, and thoughts on what supports would be helpful between appointments. Quantitative questions with Likert-style responses (ranging from 1=lowest to 5=highest) were used to rate 9 mock screens for usefulness, understandability, and importance for each view. Mock screens had been iterated after the discover phase; therefore, patient feedback during this second round further validated and helped refine the designs.

##### Generalizability to Other High-Risk Clinical Contexts

To ensure that the technological build was not *overdesigned* for MS and to support the scalability of the tool to other clinical settings, interviews were expanded to intended users in other clinical specialties associated with falls, including geriatrics, orthopedics, neurorecovery (after stroke or traumatic brain injury), and Parkinson disease (PD). Clinicians from each discipline and patients with PD were interviewed. Interview protocols used during the discover phase were adapted to reference specific disciplines and diseases, whereas the questions (qualitative and quantitative) remained the same to yield a parallel assessment of each audience’s experiences, preferences, capabilities, opportunities, and motivations.

##### Analysis

Qualitative and quantitative interview analysis used the same inductive coding and calculation techniques, respectively, used during the discover phase. The results were analyzed by the interviewer, with ongoing thematic consultation with the stakeholder advisory team, and used to inform and prioritize design and content iterations.

#### Phase 4: Deliver

##### Stakeholder Advisory Team

The core team met with stakeholders on an ad hoc small-group basis during this phase to plan observation and tool-scoring protocols, specifically to identify a subset of questions from the Health IT Usability Evaluation Scale (Health-ITUES) derived from the Health-ITUEM to assess the 2 *subjective* components of usability—usefulness and ease of use [33]—as well as the Patient Education Materials Assessment Tool for Audiovisual Materials to assess understandability and actionability [50]. As recommended for digital tool validation [45], a single survey question—Net Promoter Score (NPS)—was asked regarding the likelihood that users (patients and clinicians) would recommend the MS-FIT to colleagues or friends. Additional conversations focused on the scalability of the tool, as well as the qualitative and quantitative feedback received.

##### Observations and Scoring

Observations and scoring for the patient-facing falls assessment survey took place with 2 audiences: people with MS and people with PD. Patients scheduled for a routine upcoming in-person clinical visit with their neurologist were contacted and invited to participate in testing and evaluating the tool. After providing informed written consent, and while being observed by the interviewer, participants were asked to engage with the MS-FIT minimum viable product consisting of the falls assessment survey and accompanying patient instructions while being observed by the interviewer. Patients were specifically asked to complete the falls assessment survey by entering up to 5 falls (real or hypothetical) that had occurred in the prior 2 weeks and responding to on-screen prompts to provide context about each reported fall. Patients could ask questions of the interviewer, if needed. After survey submission, each patient was asked to complete an 18-item survey about their experience to assess usability, usefulness and ease of use, likability, understandability, actionability, and NPS. Patients were subsequently asked if they had any feedback about their experience. Feedback was documented in field notes captured contemporaneously.

Clinicians seeing people with MS and those with PD who had just been observed entering data in the falls assessment survey were asked to launch the MS-FIT BRIDGE app in real-time clinical encounters with these patients to review the falls and contextual data the patient had entered and to engage with the various widgets designed to help evaluate and address reported falls. The interviewer met with the clinicians immediately after the encounters to conduct in-person exit interviews and administer a 9-item survey to assess usability, usefulness and ease of use, likability, understandability, actionability, and NPS. Clinicians were subsequently asked whether they had any feedback about their experience, including any barriers to use and functionality challenges. Feedback was documented in contemporaneous field notes.

##### Analysis

Qualitative feedback, although limited, was analyzed by the interviewer using the same inductive coding technique used during the previous 2 interview phases. Quantitative questions with Likert-style responses (ranging from 1=lowest to 5=highest) were used to score likability, usability, usefulness, and ease of use. Understandability and actionability were assessed using a binary *agree* or *disagree* scale. Another member of the research team entered quantitative responses into REDCap, which was used to calculate means and SDs for all Likert-style responses and total binary responses. NPS responses (0-10 scale) were calculated by the interviewer by subtracting the percentage who were detractors (those who scored 0 to 6) from the percentage who were promoters (those who scored 9 or 10). An NPS >0 was considered good, >20 was considered favorable, and >50 was considered excellent.

##### Development Action Items

Once the tool was live, the developer was able to debug MS-FIT; iterate based on patient, clinician, and stakeholder feedback; and redebug as needed.

## Results

### Overview

**Figure 4:**
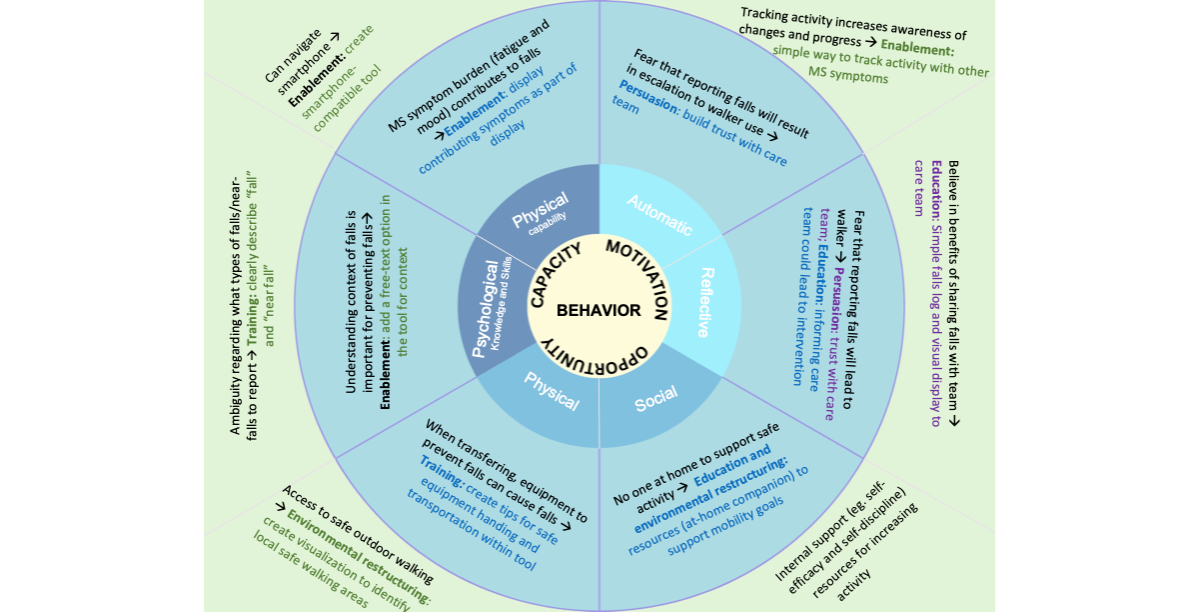
Example of mapping blockers and boosters relating to falls prevention (findings from interviews with patients with multiple sclerosis [MS]) to the Behavioral Change Wheel [49] and associated behaviorally informed intervention solution features. Examples for each of the sections of the capability, opportunity, motivation, and behavior (COM-B) model are highlighted, showing how these integrate into the Behavioral Change Wheel. The examples provided relate to patients’ reported goals, blockers (features that block falls prevention behavior), and boosters (features that boost behaviors related to falls prevention).

Demographic information about each interview panel is shown in Multimedia Appendix 1 [42]*.* Altogether, 30 patients of diverse ages, disability levels, and technological literacy as well as 14 clinicians provided at least 1 round of feedback. The level of involvement from the users ranged from testers to informants [32]. Feedback from both rounds of interviews with people with MS, MS clinician comments, and feedback from other high-risk clinical context patient and clinician interviews were integrated into the final MS-FIT design. Iterative interview feedback was categorized into activity blockers (what keeps people from performing a behavior) and boosters (what is already working well that we can build on) in the COM-B model. Examples of how interview feedback findings fit into the COM-B and BCW, along with intervention function solution features integrated into MS-FIT, are shown in Figure 4*.* Details are provided in Table 1*.* Further discussions with clinicians and patients in other high-risk clinical contexts confirmed the findings from the MS context. Across these specialties, the main barriers to falls prevention efforts included access to specialized PT (availability and physical ability to access it), insurance coverage, ability to adapt the home to improve safety, the adequate use of assistive devices, and COVID-19–related restrictions to community exercise areas.

The overview of findings from interviews with clinicians and participants with MS, highlighting areas that block or boost patient and clinician behavior change with regard to falls and falls prevention, are shown in Multimedia Appendix 2.

**Table 1:** Scoring of the final University of California San Francisco Multiple Sclerosis Falls InsightTrack app (REDCap [Research Electronic Data Capture]) by patients with multiple sclerosis: usability, ease of use, and likability (n=10).

Health-ITUES^a^–based questions for usability, ease of use, and likability	Score, mean (SD)	Score <4 out of 5, n (%)
“It is useful to report if I’ve had any falls or near falls every 2 weeks”	4.80 (0.42)	0 (0)
“It is useful to have my survey answers sent to my care team”	4.90 (0.32)	0 (0)
“The survey asks about important topics”	4.70 (0.48)	0 (0)
“I am comfortable with my ability to complete the survey”	4.80 (0.42)	0 (0)
“I find the survey easy to use”	4.80 (0.42)	0 (0)
“I can easily remember how to access the survey through my email”	4.60 (0.70)	1 (10)
“I like the survey”	4.80 (0.42)	0 (0)

^a^Health-ITUES: Health IT Usability Evaluation Scale (scores range from 1=strongly disagree to 5=strongly agree).

### Tool Components

Thematic saturation was reached after 5 patient interviews (round 1), and we incorporated these insights into prototypes for an additional 5 patient interviews (round 2), which were then iteratively reviewed.

### UCSF Support Self-Monitoring: A Patient-Facing Tool to Track Falls and Self-Monitor

#### Tool Architecture

One key and consistent theme emerging from patient interviews was a preference for a simpler design for the patient-facing tool than had been initially conceived. Combined with a goal of maintaining confidentiality and keeping personal information within our university firewall, the study team opted for a tailored REDCap app rather than a custom new app.

#### Tool Components

**Figure 5:**
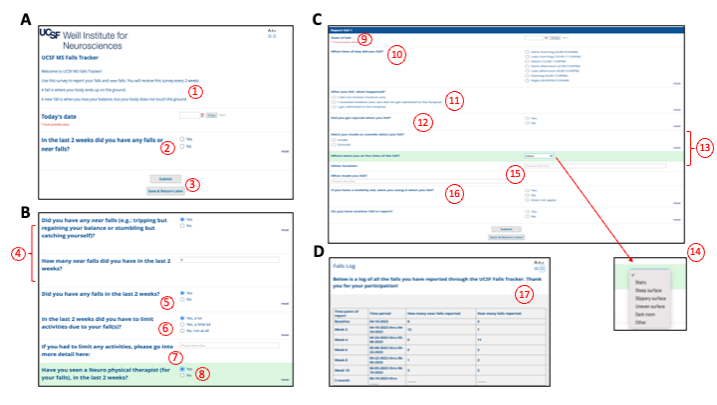
University of California San Francisco (UCSF) Multiple Sclerosis (MS) Falls Tracker: a patient-facing tool to track falls and support self-monitoring. This is the “MS-FIT [Multiple Sclerosis Falls InsightTrack] patient survey V2.0,” sent via email to patients with a REDCap (Research Electronic Data Capture) survey link. Key features mapping to user-generated perspectives and feedback and to the capability, opportunity, motivation, and behavior (COM-B) framework are denoted by a red number and described in Textbox 1.

Key features informed by patient and clinician feedback are detailed in Figure 5. Key features mapping to the COM-B framework (Multimedia Appendix 2 and Figure 4) are denoted by a red number and described in Textbox 1.

**University of California San Francisco Multiple Sclerosis Falls InsightTrack: key features. The numbers correspond to the red numbers in Figure 5, which denote key features mapping to the capability, opportunity, motivation, and behavior (COM-B) framework.**

**Concise and precise falls screening**

1. Clear definitions were preferred to distinguish between a fall and a near fall to support the reporting of meaningful data.
2. An easy-to-use and simple 1-question tool that could be completed frequently (every 2 wk) was preferred to relying on “flawed memory” to report falls during sporadic clinic visits: if “No,” then the survey ends at this point; if “Yes,” then branching logic continues.
3. The ability to easily report each fall or near fall separately was preferred. The ability to edit (return later) was important for reducing burden.
4. Simple reporting for near falls (yes or no and overall number) was preferred, given the large volume of near falls experienced by some patients and the potential burden and time commitment of providing details.
5. The 2-wk epoch between reporting was determined feasible (balance between memory and overburdening).
6. The ability to report activity limitations was preferred because these pertain to primary goals with regard to the “ability to continue independence for activities of daily living” and to “stay active.”
7. Because of the heterogeneity in answers, a free-text option would allow patients to add further details regarding activity limitations.
8. Indicating whether the patient has seen a neurorehabilitation specialist could help clinicians triage the continued plan of care.

**Detailed context of falls (optional)**

**Figure 6:**
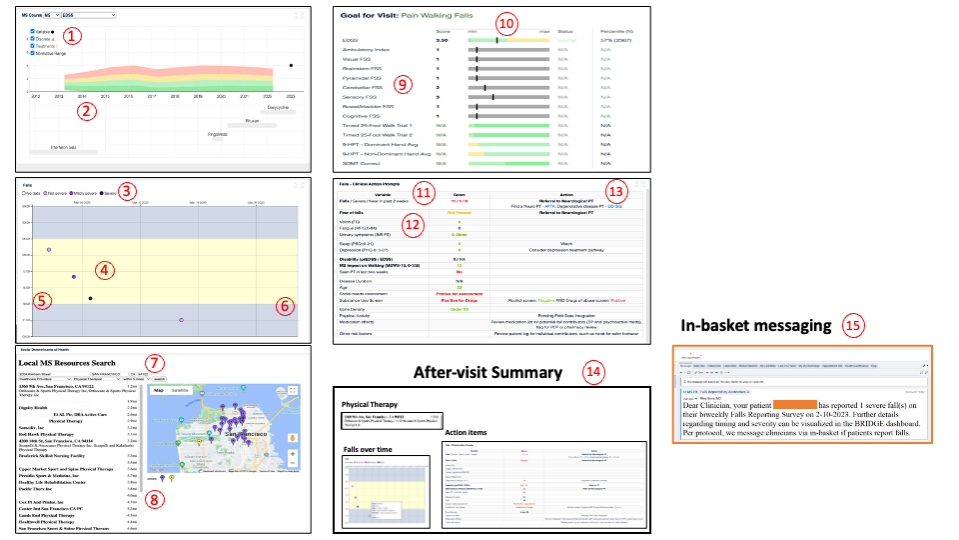
Multiple Sclerosis Falls InsightTrack (MS-FIT) clinical management dashboard, integrated into the Epic electronic health record (Epic Systems Corporation). This is the “MS-FIT BRIDGE version 3.0,” which is viewable from Epic in the electronic health record at the time of the clinic visit. Key features mapping to user-generated perspectives and feedback and to the capability, opportunity, motivation, and behavior (COM-B) framework are denoted by a red number and described in Textbox 2.

1. Recording the date of the fall using a simple button allows the tool to display each fall into the longitudinal representation (refer to Textbox 2; Figure 6).
2. The time of falls can also inform falls context (eg, in the dark or when fatigued). The 24-h day was divided into time blocks for clarity and to reduce recall error of exact time.
3. Information regarding the medical consequences of a fall can inform both its severity and the clinical follow-up needed.
4. Injury after a fall is considered distinct from seeking and receiving medical attention.
5. Fall location can inform prevention efforts, including home safety; “some falls inside the home can be avoided through modifications such as removing a rug, better lighting etc.”
6. Other details of the fall location can also inform home safety and prevention (eg, curb, stairs, and poor lighting).
7. Specifying whether falls occur because of factors related to multiple sclerosis or other factors (obstacle, etc) is important owing to the heterogeneity of fall triggers and of clinical responses.
8. The question “If you have a mobility aid, were you using it when you fell?” can remind patients to use the assistive device and can cue clinicians of the need to modify or change the current assistive device.
9. A falls log is provided to patients and shows the reported falls over time.

**Closing the loop: real-time in-basket messaging**

1. Enabling the reporting (patient) and ascertainment (clinician) of falls at regular intervals optimizes timeliness (vs periodic visits) while maintaining low burden (vs daily or “at time of fall”). If a severe fall is reported on the biweekly survey, an in-basket message to the electronic medical record alerts the care team in a manner integrated into the clinical workflow (Figure 6, #15).

**Multiple Sclerosis Falls InsightTrack clinical management dashboard integrated into the Epic electronic health record: key features. The numbers correspond to the red numbers in Figure 6, which denote key features mapping to the capability, opportunity, motivation, and behavior (COM-B) framework.**

Longitudinal multiple sclerosis trajectory widget (visualizes patients’ disease and medication trajectory over time with integrated normative ranges)

1. Ability to toggle through disability measures (eg, Expanded Disability Status Scale [EDSS] and Timed 25-Foot Walk)
2. Succinct overview of patient’s longitudinal MS trajectory, including relapses, disability, medications, and normative data

Longitudinal falls widget (visualizes falls reported every 2 wk by the patient using their patient-facing app [Figure 5] data regarding date, time, and severity of each fall on 1 display)

1. Fall severity visualized by color shade (grading falls by severity considered important to trigger an alert to the care team and to inform type of clinical response)
2. Ability to include a way to visualize the falls log with falls over time
3. Estimated time of day of the fall can inform further interventions needed, including vision check, home safety evaluation, and medication review (especially for Parkinson disease)
4. Time of day visualized with colors for daytime (lighter: yellow) and nighttime (darker: blue) preferred by all stakeholders

Community resources widget (map automatically displays the patient’s home community and allows for web-based identification of MS health care professionals in their community)

1. Automated display of MS professionals (physical therapist, occupational therapist, and talk therapist) in the patient’s community, which reduces barriers for patient to identify local recourses once physical therapy or other referrals have been placed
2. Contact information and driving navigations between the patient’s home and the resources automated, can be pasted into the patient’s after-visit summary or the clinician’s note

Cross-sectional widget (summary display with 2 tabs displaying clinical disability outcomes and patient-reported outcomes [PROs]; clinician can toggle between time points)

1. Clinic-based performance measures (walking speed, hand function, and cognition) and disability outcomes (EDSS with separate functional system scores) as well as PROs can inform a more global assessment of the patient at given time point
2. Color-coded normative ranges can provide rapid assessment of whether patient’s given function is within “normal” range

Falls treatment– and action-prompt widget (tabulates core data needed for a comprehensive assessment of falls risk and prevention; for each category, patient’s score is colored according to severity, and possible action prompts are displayed)

### Phase 4: Deliver

Altogether, 15 patients (10 with MS and 5 with PD) with an age range of 34 to 79 years and 6 MS clinicians with a clinical experience range of 2 to 22 years (Multimedia Appendix 1) launched the tool components *live* and provided feedback.

#### Patient-Facing UCSF MS-FIT

##### People With MS

Of the 15 patients, 10 (67%) had been diagnosed with MS; they had a mean age of 48.8 (SD 8.8; range 34-60) years, with disability level (EDSS score) ranging from 1.5 to 6.0 and a median disease duration of 14.5 (IQR 6.3-24; range 2-27) years. The feedback from people with MS was overwhelmingly positive (Table 1). Likability scores were all NPS≥100 (all promoters). The survey was found to be brief and clear. Patients appreciated the benefit of the closed-loop system and the overall impact on clinical encounters.

##### Patients With PD

Of the 15 patients, 5 (33%) had been diagnosed with PD; they had a mean age of 60.6 (SD 13.2; range 46-79) years, with a median disability level (Unified Parkinson’s Disease Rating Scale score) of 31 (IQR 30.3-8.5; range 17-42) and a median disease duration of 4 (IQR 1.5-8.5, range 1-10) years. Overall, the NPS was found to be 0 (20%-20%, with 1/5, 20% detractor, 1/5, 20% promoter, and 3/5, 60% passive scores that trended toward promoters), indicating that patients with PD could be easily swayed to use MS-FIT. The mean scores on the Health-ITUES questions were all >4 (ie, agree or strongly agree), and only 1 score was <3 out of 5 (Table 2).

Qualitative insights from the interviews revealed that *falling*, *fear of falling*, and *thinking about falling* were “not at the top of their list,” in contrast to people with MS. Nevertheless, patients with PD found the tracker “easy to fill out,” and they “liked the idea of reporting falls and reporting if [they] experienced fear of falling.” Patients with PD felt that it was important to have the ability to increase the font size (incorporated into MS-FIT patient survey v 2.0; Figure 5).

For future use in PD, patients reported that it would be important for ease of use and usability to have the ability to report motor vehicle accidents and specific PD symptoms as they relate to falls risk. Patients with PD also reported greater issues with using an iPad (motor or tremor issues).

**Table 2:** Scoring of the final University of California San Francisco Multiple Sclerosis Falls Tracker (REDCap [Research Electronic Data Capture]) by patients with Parkinson disease: usability, ease of use, and likability (n=5).

Health-ITUES^a^–based questions for usability, ease of use, and likability	Score, mean (SD)	Score <4 out of 5, n (%)
“It is useful to report if I’ve had any falls or near falls every 2 weeks”	4.40 (0.89)	1 (20)
“It is useful to have my survey answers sent to my care team”	4.20 (1.22)	1 (20)
“The survey asks about important topics”	4.00 (1.00)	2 (40)
“I am comfortable with my ability to complete the survey”	4.60 (0.89)	1 (20)
“I find the survey easy to use”	4.60 (0.89)	1 (20)
“I can easily remember how to access the survey through my email”	4.20 (1.10)	2 (40)
“I like the survey”	4.20 (1.10)	2 (40)

^a^Health-ITUES: Health IT Usability Evaluation Scale (scores range from 1=strongly disagree to 5=strongly agree).

#### MS-FIT Clinical Management Dashboard

Overall, the MS clinicians (n=6) rated the dashboard highly (NPS=16.67; Table 3):

I like that [the app] summarizes important clinical information in an easily digestible format, and the new widget that includes an MS [multiple sclerosis]-specific review of systems and actionable items seems like it will help ensure well-rounded MS care!

Clinician 1

With regard to reporting falls and near falls, the MS clinicians noted multiple benefits to aiding with patient care:

You can infer a lot from [fall data] in terms of disease activity, disease course, changes in a patient’s life, their living setting, their support. If you see a jump in falls or the onset of falls in a patient who wasn’t falling—it is worthy of clinical attention and needs to be addressed. It would give us an objective way to know if interventions are helping to reduce falls.

Clinician 2

Near falls are particularly underscreened, so any granularity on near falls would be helpful.

Clinician 3

For some patients, near falls may not be worth reporting—may just be part of life. But other patients it could make sense for. Any change from baseline has potential to be significant. Near falls can be [a] canary in a coal mine.

Clinician 5

**Table 3:** Scoring of the final University of California San Francisco Multiple Sclerosis Falls BRIDGE dashboard by multiple sclerosis clinicians: usability, ease of use, and likability.

Health-ITUES^a^–based questions for usability, ease of use, and likability	Score, mean (SD)	Score <4 out of 5, n (%)
“The information that appears in BRIDGE is useful to me.”	4.80 (0.41)	0 (0)
“It is useful to be updated on my patient’s significant fall activity between appointments.”	4.50 (0.84)	1 (17)
“I find BRIDGE easy to use.”	4.20 (0.75)	1 (20)
“I can always remember how to access BRIDGE.”	4.00 (1.10)	1 (20)
“I like BRIDGE.”	4.50 (0.55)	0 (0)

^a^Health-ITUES: Health IT Usability Evaluation Scale (scores range from 1=strongly disagree to 5=strongly agree).

## Discussion

### Principal Findings

To our knowledge, this is the first tool designed using the HCD framework, anchored in the COM-B approach to behavior change, and capable of delivering relevant information at the point of care in line with the *5 rights* with the aim of preventing falls in people with MS. Other apps have been developed, although the focus has mainly been on 1 component of falls (eg, evaluating falls risk [51]) at a time. In addition, many large-scale clinical research projects, such as those conducted at the Stanford Center for Digital Health and the Remote Assessment of Disease and Relapse–Central Nervous System program, are exploring applications of wearable data. However, most of the collected wearable data remain inaccessible for visualization or integration within a clinic’s EHR. This limitation can impede the effective use of PGD by clinicians and compromise patient-physician collaboration related to PGD [52]. MS-FIT fills a critical gap in multimodal closed-loop self-management apps for falls monitoring and prevention.

Through extensive stakeholder engagement, MS-FIT offers novel aspects of customization, generalizability, and scalability, integrating multiple data streams relevant to reducing falls. It provides rapid personalized in-basket notifications, limited to severe falls, and digitally displays PGD through the EHR, increasing the likelihood of adoption by patients and clinicians.

Designed in collaboration with patients and clinicians, MS-FIT has emerged as a well-received closed-loop tool for tracking falls and reducing falls risk in individuals with MS. Patients liked its brevity, simplicity, and overall utility, recognizing its potential to enhance clinical discussions. The utility of between-visit reporting and contextualized information for identifying modifiable falls risks was acknowledged by both patients and clinicians. The trial phase aims to validate its low-burden design in practice. Clinicians welcomed the closed-loop system, foreseeing proactive interventions and streamlined implementation. Longitudinal falls visualization, incorporating time and severity, along with clinician prompts targeting MS symptoms and medication effects, was favored for its ability to capture often overlooked components during regular visits.

Another noteworthy finding was the minimal number of interviews required to attain thematic saturation in our initial discover phase, indicating that some clear guidance for potentially high-value initial design features was achieved with a minimal sample size. This could be due to the fact that MS-FIT was based on an initial prototype developed during a prediscover phase using patient and clinician feedback. It could also be attributed to homogeneous samples of study participants consulted throughout the discover and design phases. Overall design efficiency was likely aided by the experience and regular input of interprofessional teams.

The ongoing process involves testing MS-FIT in a prospective longitudinal study in a cohort of 100 adults with MS over 12 months. The primary objectives of this larger study include assessing the adoption rate of the tool, evaluating the level of sustained use of the tool, monitoring adherence to falls reporting, and assessing study retention over the 12-month period. Secondary and exploratory analyses will center around the prediction of adoption, sustained use, adherence to action prompts, and study retention. To determine effectiveness, the study will compare in-study falls with a prior falls data set (Fitbit remote monitoring in MS) [41], and patient satisfaction will be assessed during an exit interview.

### Scalability

Our approach, characterized by the selection of key technological and clinical features, allows for the scalability and generalizability of the tool’s modular infrastructure to various symptoms, conditions, and clinical settings for other high fall-risk diseases as well as other symptoms within MS (eg, bladder dysfunction). Technological factors for scalability include (1) high-quality, widely shareable static visualizations; and (2) optimized industry standards for code sharing with clinicians in other health care settings, such as other MS centers using Epic EHR. However, successful integration into other health systems depends on the internal governance and motivation within each system.

### Limitations

All interviews were conducted remotely, using the UCSF Zoom video platform, which may have biased the patient stakeholders to people who are technologically literate and have access to the internet. However, 92% of people in the United States have access to the internet [53], and given that MS-FIT is an app, users (patients or caregivers) are expected to possess a certain level of technical proficiency. Only clinicians at UCSF and patients seen by this (broad) group of clinicians were interviewed; therefore, we may have missed important feedback from a wider cohort of users. Although HCD is favored for user-driven eHealth innovations, certain limitations exist [32], including a narrow focus; thus, exploring alternatives such as value-sensitive design, citizen science, and more-than-human design could enhance inclusivity and impact within eHealth innovation [54]. Finally, having the interviewer serve as the primary coder could have introduced bias into the qualitative analysis process. Stakeholder advisory group engagement in the coding process was an effort to reduce any potential bias.

### Conclusions

MS-FIT delivers relevant data to clinicians through an embedded window within the EHR, following the *5 rights* approach. By using MS-FIT for data processing and algorithms, we reduce clinician burden while enhancing care. Our innovation extends to enabling and integrating real-world PGD as well as clinical and community-level factors, providing actionable information to empower self-care and addressing the impact of falls in people with MS. Our preliminary data indicate that this tool and design extend beyond MS and can be applied to other conditions associated with falls as well as the fear of falls and their associated consequences. To test the feasibility and effectiveness of the app, a clinical trial is ongoing (University of California San Francisco Clinical Trials identifier: NCT05837949).

## Appendix

Multimedia Appendix 1 Demographic information for each round of interviews that led to the development of Multiple Sclerosis Falls InsightTrack.

Multimedia Appendix 2 Overview of findings from interviews with clinicians and participants with multiple sclerosis (MS), highlighting areas that block or boost patient and clinician behavior change with regard to falls and falls prevention. The table indicates whether intervention solution features were incorporated into the Multiple Sclerosis Falls InsightTrack (MS-FIT) patient survey, the clinician dashboard, or both.

## Abbreviations

API: application programming interface
BCW: Behavioral Change Wheel
COM-B: capability, opportunity, motivation, and behavior
CSS: cascading style sheets
EHR: electronic health record
FHIR: Fast Healthcare Interoperability Resources
HCD: human-centered design
Health-ITUEM: Health IT Usability Evaluation Model
Health-ITUES: Health IT Usability Evaluation Scale
MS: multiple sclerosis
MS-FIT: Multiple Sclerosis Falls InsightTrack
PD: Parkinson disease
PGD: patient-generated data
PT: physical therapy
REDCap: Research Electronic Data Capture
SMART: Substitutable Medical Apps and Reusable Technologies
UCSF: University of California San Francisco

## Data Availability

The data sets generated and analyzed during this study are available from the corresponding author on reasonable request.
