# Supplementary material for: A Closed-Loop Falls Monitoring and Prevention App for Multiple Sclerosis Clinical Practice: Human-Centered Design of the Multiple Sclerosis Falls InsightTrack
Source: JMIR Hum Factors. 2024 Jan 11;11:e49331. doi: 10.2196/49331 (PMC10811573; doi:10.2196/49331)
Supplement: Multimedia Appendix 1 [file humanfactors_v11i1e49331_app1.docx]

## Multimedia Appendix 1

## Supplementary Table 1.

### Demographic information for each round of interviews that led to the development of MS-FIT.

### LEGEND:

| **Design Phase** | **Interview Dates** | **Interview**  (cohort, round #) | **Clinical Context** | **Sample size**  (N) | **Age range/ clinical experience** (yrs) | **Sex** (F: N, %) |
| --- | --- | --- | --- | --- | --- | --- |
| **I.** Discover | Mar ‘22 | Patients, Round 1 | MS | 5 | 49-71* | 4 (80) |
| **I-II.** Discover and Define | Apr ‘22 | Clinicians (N/A) | (MS) | 3 | 9-17* | 2(67) |
| **II-III.** Define and Develop | July ‘22 | Patients, Round 2 | MS | 5 | 39-71 | 3 (60) |
| **II-III.** Define and Develop | Dec ‘22 | Clinicians (N/A) | Generalizability  (2: Orthopedics, 1: PD, 1: Geriatrics, 1: Neurorecovery/ stroke) | 5 | N/A | 2 (40) |
| **II-III.** Define and Develop | Jan ‘23 | Patient,  Round 1 | Generalizability (PD) | 5 | 46-79 | 1 (20) |
| **IV.** Deliver | Jan ’23 | Patient (N/A) | MS | 10 | 34-63 | 10 (100) |
| **IV.** Deliver | Jan ’23 | Clinicians (N/A) | (MS) | 6 | 2-22* | 5 (83) |
| **IV.** Deliver | Feb ’23 | Patient (N/A) | Generalizability | 5 | 46-79 | 10 (100) |

Thematic saturation^42^ (where no new concepts emerged) was reached after five interviews with MS patients (round 1). As a result, for reporting purposes, the information gained from the Discover and Define Phases were merged.

* = Regardless of the range of experience, significant consistency in responses led to *thematic saturation.*^42^

Abbreviations**:** MS = multiple sclerosis, Mar = March, Apr = April, Jan = January, Feb = February, ’22 = 2022, ’23 = 2023, F = female, N/A = not applicable.
