# Supplementary material for: A Closed-Loop Falls Monitoring and Prevention App for Multiple Sclerosis Clinical Practice: Human-Centered Design of the Multiple Sclerosis Falls InsightTrack
Source: JMIR Hum Factors. 2024 Jan 11;11:e49331. doi: 10.2196/49331 (PMC10811573; doi:10.2196/49331)
Supplement: Multimedia Appendix 2 [file humanfactors_v11i1e49331_app2.docx]

| Sources of behavior | | | | | | | | | | | | | | | | | | | | Intervention functions: solution features integrated into MS-FIT | | | | | Patient survey (P), clinician dashboard (C), or both (B) | | |
| --- | --- | --- | --- | --- | --- | --- | --- | --- | --- | --- | --- | --- | --- | --- | --- | --- | --- | --- | --- | --- | --- | --- | --- | --- | --- | --- | --- |
| **Capability** | | | | | | | | | | | | | | | | | | | | | | | | | | | |
|  | | | | | | | | | | | | | | | **Physical capability** | | | | | | | | | | | | |
|  | | | | | | | | | | | | | | |  | | | **Boosters** | | | | | | | | | |
|  | | | | | | | | | | | | | | |  | | |  | | - Tools: mobility equipment helps maintain activity, regardless of EDSS^b^ score. - “Are you able to walk?” - All 5 patients report walking with the aid of equipment. | - Enablement: include all levels of MS ambulatory disability. | | | | | | B |
|  | | | | | | | | | | | | | | |  | | |  | | - Knowledge: patients can navigate smartphone. - “How confident are you using a smartphone?” - On a scale ranging from 1 to 5 (5=very confident), the average response was 4.6. | - Enablement: create smartphone-compatible tool. | | | | | | P |
|  | | | | | |  | | | | | | | | | **Blockers** | | | | | | | | |  |  |  |  |
|  | | | | | | | |  | | | |  | | | - Activity is limited by MS symptoms (ie, fatigue) or transportation (not driving). - “I am not able to drive. I need my husband’s help, but he works.”[Patient 1] - “It’s easy to wear myself out and lose my balance.” [Patient 2] | | | | | - Enablement: tool can capture and visualize even low functional mobility. | B | | | | | |  |
|  | | | | | | | | | | | | | | |  | | |  | | - MS symptom burden (fatigue and mood) contributes to falls. - “Fatigue at the end of the day gets in my way.” - “It gets irritating. I try to remain resilient, but I feel like I’m missing out.” | - Enablement: display the contributing symptoms as part of the display. | | | | | | C |
|  | | | | | | | **Psychological (knowledge and skills)** | | | | | | | | | | | | | | | | | | | |  |
|  | | | | | | |  | | | | | | | **Boosters** | | | | | | | | | | | | |  |
|  | | | | | | | | | | | | | | |  | | |  | | - Knowledge: distinguish between falls and near falls. - All 5 patients report the ability to distinguish what counts as a “fall” versus “near fall.” | - Education: define the terms in the falls reporting tool. | | | | | | B |
|  | | | | | | | | | | | | | | |  | | |  | | - Knowledge: understanding the context of falls is important for preventing falls. - “Mentally I keep a note of falls.” - “I know it is usually due to losing my balance or a foot drop.” - “I fall when walking up hills or on rough terrain.” - “It’s not just about steps—context really matters.” | - Enablement: add a free-text option in the tool for context. | | | | | | P |
|  | | | | | | | | | | | | | | |  | | |  | | - Familiar skill: patients are accustomed to reporting falls when asked.   ○ All 5 patients report being asked about falls activity during appointments. | - Enablement: create a tool to consistently prompt about falls. | | | | | | B |
|  | | | | | | | | | | | | | | |  | | |  | | - Confidence: patients have a high degree of habit and confidence in smartphone use to communicate with the care team.   ○ All 5 patients report having smartphone apps related to health; 4 (80%) of 5 report using app to track progress on a health related goal; and 4 (80%) of 5 report being “very confident” using their smartphone to communicate with the care team. | - Enablement: create an easy way to report falls. | | | | | | P |
|  | | | | | | | | | | | | | | |  | | | | **Blockers** | | | | | | | |  |
|  | | | | | | | | | | | | | | |  | | |  | | - Knowledge: there is ambiguity regarding what types of falls/near falls to report. - “Need to understand the definition of a near fall.” - “Want a very clear definition about fall versus near fall.” | - Training: clearly describe “fall” and “near fall.” | | | | | | P |
|  | | | | | | | | | | | | | | |  | | |  | | - Understanding: patients are only reporting severe falls to the care team (often not to the MS care team). - “How important is letting your care team know when you’ve fallen or almost fallen?”   ○ On a scale ranging from 1 to 5 (5=very important), the average response was 3.4.   - “When do you inform your care team?” - “When I hurt myself.” - “Seldom, if I don’t harm myself.” | - Education: informing the care team could reduce time to intervention after a fall. | | | | | | B |
|  | | | | | | | | | | | | | | |  | | |  | | - Recall: patients rely on flawed memory to report falls.   ○ All 5 patients report relying on memory when reporting falls activity during appointments. | - Enablement: use the tool to track falls, as well as the context around falls, in real time to reduce recall burden. | | | | | | B |
|  | | | | | | | | | | | | | | |  | | |  | | - Unfamiliar skill: patients are unaccustomed to using an app for disease treatment and care team interaction. - Of the 5 patients, 2 (40%) report using an app to make a decision about how to treat an illness or have a discussion with the health care provider. | - Enablement: create alerts after a fall for care team engagement. | | | | | | P |
| **Opportunity** | | | | | | | | | | | | | | | | | | | | | |  |  |  |  |  |  |
|  | | | | | | | | | | | | | | | | | **Physical opportunity** | | | | | | | | | |  |
|  | | | | | | | | | | | | |  | | | **Boosters** | | | | | | | | | | |  |
|  | | | | | | | | | | | | | | |  | | |  | | - Tools: expert-recommended mobility tools prevent falls. - All 5 patients report receiving advice from their care team about how to prevent falls; they have and use mobility tools personally. | - Training: provide safe equipment handling and transport tips. | | | | | | B |
|  | | | | | | | | | | | | | | |  | | |  | | - Access: it is important to have access to safe outdoor walking. - Of the patients who were not wheelchair bound (3/5, 60%), all reported having a place to walk outside of the home. | - Environmental restructuring: create visualization to identify local safe walking areas. | | | | | | B |
|  |  | **Blockers** | | | | | | | | | | | | | | | | | | | | | | | |  |  |
|  | |  | | | |  | | - Tools: when transferring, equipment to prevent falls can cause falls. - Using poles and transferring scooter in/out of the car was cited as causing near falls. | | | | | | | | | | | | - Education: create tips for safe equipment handing and transportation within the tool. | | | | | | | C |
|  | | | **Social environment** | | | | | | | | | | | | | | | | | | | | | | | |  |
|  | | |  | | | | | | | | **Boosters** | | | | | | | | | | | | | | | |  |
|  | | | | | | | | | | | | | | |  | | |  | | • Support: for increasing activity, it is important to have internal (ie, self-efficacy and self-discipline) resources as well as external (ie, partner and care team) resources.   - Of the 5 patients, 4 (80%) report having the support or encouragement to be as active as desired. | - Enablement: create a simple activity tracker log with a streak feature, which is shareable. | | | | | | B |
|  | | | | | | | | | | | | | | |  | | | **Blockers** | | | | | | | | |  |
|  | | | | | | | | | | | | | | |  | | |  | | - Support: COVID-19–related restrictions hampered mobility. - The pandemic was cited as a barrier to activity. | - Enablement/environmental restructuring: offer a PT^c^ referral to home exercises. | | | | | | B |
|  | | | | | | | | | | | | | | |  | | |  | | • Support: no one at home to support safe activity.   - The lack of a daytime caregiver was cited as a barrier to activity. | - Education/environmental restructuring: create resources (at-home companion) to support mobility goals. | | | | | | P |
| **Motivation** | | | | | | | | | | | | | | | | | | | | | | |  |  |  |  |  |
|  | | | | | | | | | | | | | | | **Reflective** | | | | | | | |  |  |  |  |  |
|  | | | | | | | | | | | | | | |  | | | **Boosters** | | | | | | | | |  |
|  | | | | | | | | | | | | | | |  | | |  | | - Believe in the benefits of sharing falls with the care team. - “If I’m falling more often, they need to know. Something new may be happening.” - “Tracking would be very good—it’s hard to remember.” | - Education: create a simple falls log and visual display to the care team. | | | | | | B |
|  | | | | | | | | | | | | | | |  | | |  | | - Personal responsibility around falls prevention requires daily management. - “I pay more attention to walking and fatigue. Change how I do stairs.” - “Slowing down my thinking to match my body’s ability.” | - Enablement: create an easy way to stay mindful of falls risk and strategies to prevent falls. | | | | | | B |
|  | | | | |  | | | | | **Blockers** | | | | | | | | | | | | | | | | |  |
|  | |  | | | |  | | - Fear of falling reduces mobility. - “I have a tendency to not move and am less mobile as a result.” - “I’m not afraid, just more cautious; maybe more hesitant. Nervous about equipment.” | | | | | | | | | | | | - Education: informing the care team could lead to treatment adjustment. | | | | | | | C |
|  | |  | | | |  | | - There is fear that reporting falls will lead to walker. - “My neurologist isn’t focused on my staying mobile—they’re more focused on my safety. I want to stay at this stage.” - “My neurologist suggests using a wheelchair to protect myself.” - “I want to stay mobile for as long as possible.” | | | | | | | | | | | | - Persuasion: trust with the care team. - Education: informing the care team could lead to intervention adjustment. | | | | | | | P |
|  | |  | | | |  | | - Slowing down to avoid falls means getting less done during the day. - “It’s irritating. Plays on my psyche. I try to stay positive and try to remain resilient, but I feel like I’m missing out.” - “I’m always aware that activities are challenging. I can't cook—nothing steady to hold on to in the kitchen. Sometimes I just don’t have enough physical and emotional energy.” | | | | | | | | | | | | - Education: educate patients on going slow to stay mobile. - Enablement: ensure that falls reporting is quick and easy. | | | | | | | B |
|  | | | | **Automatic** | | | | | | | | | | | | | | | | | | | | | | |  |
|  | | | |  | | | | | **Boosters** | | | | | | | | | | | | | | | | | |  |
|  | | | | | | | | | | | | | | |  | | |  | | - Patients believe that preventing falls and staying active is beneficial. - “I want to be able to keep walking. When I was diagnosed, I was told I’d need a cane, then a walker, then a wheelchair. Tracking my activity equals progress.” - “If I can’t reduce falls, I can’t accomplish things.” | - Education: create a simple tool to prompt interventions to prevent falls. | | | | | | B |
|  | | | | | | | | | | | | | | |  | | |  | | - Tracking activity increases patients’ awareness of changes and progress. - “I want to be aware of big changes. If I’m doing more, it’s encouraging, but I don’t want to obsess.” - “It’s beneficial if they see patterns in data that I don’t see. Not all falls are the same.” | - Enablement: create a simple way to track activity with other MS symptoms. | | | | | | B |
|  | | | | | | | | | | | | | | |  | | |  | | - Patients believe that higher level of activity can protect against comorbidities. - “Tracking how active I am is very important. I’m aware that I need to move more, increase my heart rate for health.” | - Environmental restructuring: create reinforcement or little reminders that being active helps with MS/overall health. | | | | | | B |
|  | | | | | | | | | | | | | | |  | | | **Blockers** | | | | | | | | |  |
|  | | | | | | | | | | | | | | |  | | |  | | - Patients believe that the care team has limited influence to reduce falls. - “They’re not in the situation I’m in.” - “It’s just a part of having MS. I don’t see how it [falls reporting] does or doesn’t affect it.” | - Environmental restructuring: informing the care team could lead to therapy adjustment. | | | | | | B |

**Legend**: Overview of findings from interviews with clinicians and participants with multiple sclerosis (MS), highlighting areas that block or boost patient and clinician behavior change with regard to falls and falls prevention. The table indicates whether intervention solution features were incorporated into the Multiple Sclerosis Falls InsightTrack (MS-FIT) patient survey, the clinician dashboard, or both.^a^

^a^These findings are summarized according to the capability, opportunity, motivation, and behavior (COM-B) framework and the Behavioral Change Wheel. Insights from the interviews are organized into what features boost or block behaviors related to falls prevention (the change) and how these features might be integrated into the MS-FIT design.

^b^EDSS: Expanded Disability Status Scale.

^c^PT: physical therapy.
